# Effects of Exogenous Hydrogen Sulfide on Diabetic Metabolic Disorders in db/db Mice Are Associated With Gut Bacterial and Fungal Microbiota

**DOI:** 10.3389/fcimb.2022.801331

**Published:** 2022-03-29

**Authors:** Jian Liu, Wei Zhao, Zi-Wei Gao, Ning Liu, Wei-Hua Zhang, Hong Ling

**Affiliations:** ^1^ Department of Microbiology, Harbin Medical University, Harbin, China; ^2^ Department of Pathophysiology, Harbin Medical University, Harbin, China; ^3^ Wu Lien-Teh Institute, Harbin Medical University, Harbin, China; ^4^ Heilongjiang Provincial Key Laboratory of Infection and Immunity, Harbin, China; ^5^ Key Laboratory of Pathogen Biology, Harbin, China

**Keywords:** exogenous H_2_S, type 2 diabetes, db/db mice, metabolic disorders, gut microbiota

## Abstract

The effects of hydrogen sulfide (H_2_S) on diabetic metabolic disorders are still controversial, and the mechanisms underlying these effects remain largely unknown. This study was conducted to investigate the potential relationship between the gut microbiota and the improvement of diabetic metabolic disorders by exogenous H_2_S in obese db/db mice. The db/db mice were treated with sodium hydrosulfide (NaHS) (80 μmol/kg), or vehicle for 16 weeks, respectively. We measured the serum H_2_S, obesity parameters, glucose homeostasis, and triglyceride. The sequencing of bacterial 16S rRNA gene and fungal internal transcribed spacer (ITS) in the cecal contents of NaHS-treated mice was performed to evaluate the gut microbial communities. We found that supplying exogenous H_2_S for 16 weeks significantly inhibited the increase of serum triglyceride, blood glucose, and insulin levels and altered specifically the gut bacterial microbiota structure in db/db mice. The relative abundance of some bacterial genera was correlated with the H_2_S or blood glucose level. Indeed, exogenous H_2_S increased Firmicutes and decreased Bacteroidetes at the phylum level along with changes of abundance of multifarious genera. Among them, *Unclassified_Enterobacteriaceae*, *Prevotella*, and *Lactobacillus* decreased and *Unclassified_Ruminococcaceae*, *Oscillospira*, *Ruminococcus*, *Sutterella*, and *Desulfovibrio* increased. For fungi, exogenous H_2_S decreased the abundance of *Candida* and *Aspergillus*. Here we demonstrated that, in diabetes, microbial dysbiosis may not be just limited to bacteria due to the inter-linked metabolic interactions among bacteria and fungi in the gut. The beneficial effects of exogenous H_2_S on diabetic metabolic disorders are likely associated with the alterations of specific microbiota.

## Introduction

Recently, increasing evidence strongly supports that the abnormal composition of gut microbiota is closely associated with obesity or diabetes (Scheithauer et al., 2020; Arora et al., 2021; Fang et al., 2021). Meanwhile, probiotics (Kashiwagi et al., 2021), prebiotics (Zheng et al., 2018), and dietary supplements (Chen et al., 2019; Li et al., 2020) have been used in animal studies to assess the effects of altered gut microbiota on obesity and type 2 diabetes.

Leptin receptor-deficient mice (Db/db) mice have been reported with microbiota disorders showing that Firmicutes decreased significantly in abundance, which is the most abundant phylum in db/m mice. Several bacterial taxa, including Lactobacillus and some Bacteroides, were less abundant, and *Akkermansia muciniphila* was more abundant in db/db mice (Singh et al., 2020). In addition, sulfur-containing metabolites are significantly altered and play a key role in type 2 diabetes metabolism in db/db mice (Walker et al., 2014).

Hydrogen sulfide (H_2_S), as a new gasotransmitter in the body, plays an important role in diverse physiological functions, such as antioxidative stress, anti-inflammation, and anti-hypertension (Sokolov et al., 2021). Some studies have shown that H_2_S levels in the blood circulation decreased significantly in the diabetic animal models (Jain et al., 2010; Suzuki et al., 2011), obese people (Whiteman et al., 2010), and patients with type 2 diabetes (Jain et al., 2010; Whiteman et al., 2010).

To date, accumulative evidence from animal models demonstrated that supplying exogenous H_2_S donors is beneficial for the recovery of diabetes-related disorders (Wallace and Wang, 2015; Li et al., 2021; Zhang et al., 2021). Nevertheless, some studies have shown various effects of exogenous H_2_S in different models associated with metabolic disorder. In Goto-Kakizaki (GK) diabetic rats, chronic NaHS treatment (30 μmol·kg^-1^·day^-1^) decreased fasting blood glucose, increased insulin sensitivity, and increased glucose tolerance (Xue et al., 2013). Similar insulin-sensitizing effects of NaHS treatment were also observed in Wistar rats (Xue et al., 2013). Type 2 diabetes is often accompanied by obesity. Wu et al. employed a diet-induced obesity (DIO) mouse model to find that the administration of H_2_S donor NaHS could significantly recover the hepatic structure and decrease the accumulation of lipids including triglyceride and total cholesterol in high-fat diet (HFD)-induced obese mice (Wu et al., 2015). Recent studies have shown that db/db mice treated with NaHS for 10 weeks displayed improvements in glucose tolerance and serum insulin levels but did not alter the increase in body weight or serum triglyceride levels (Sun et al., 2018). However, different results were reported showing that there was no effect on blood glucose, serum lipids, and glucose tolerance in db/db mice after NaHS injection (Wu et al., 2017). Chronic administration of NaHS in particular at high doses impaired carbohydrate metabolism in type 2 diabetic rats (Gheibi et al., 2019). Consistent with this, the dose of NaHS at 120 μmol·kg^-1^·day^-1^ did not exert an insulin-sensitizing effect in GK rats (Xue et al., 2013). Taken together, the differences in diabetic model, NaHS dosage, or treatment course could be considered in influence factors of H_2_S effect on diabetes.

In recent years, it has been found that exogenous H_2_S donors appear to be able to influence the gut microbiota (Blackler et al., 2015; Motta et al., 2015; Walsh et al., 2020). For instance, exogenous H_2_S donors protect against NSAID-induced enteropathy through modulation of the microbiota which caused a significant decrease in multiple Clostridiales families, such as Ruminococcaceae and Eubacteriaceae, and an increase in abundance of *Mucispirillum* (Blackler et al., 2015). Low levels of endogenous or exogenous H_2_S directly stabilize mucus layers, prevent fragmentation and adherence of the microbiota biofilm to the epithelium, inhibit the release of invasive pathobionts, and help resolve inflammation and tissue injury (Motta et al., 2015). The cross talk between H_2_S, the gut microbiota, and health has been the topics of recent reviews (Buret et al., 2022). Other studies have reported that dietary H_2_S may also modulate the abundance and function of microbiota (Burrichter et al., 2018; Frommeyer et al., 2020). However, there are still limited data which are available to verify the effects of H_2_S on the gut microbiota. Although we may propose that exogenous H_2_S may help resolve inflammation and tissue injury in gut by stabilizing mucus layers and prevent fragmentation of the biofilm, extensive studies are needed to characterize changes in microbiome in the context of exposure to exogenous H_2_S.

Therefore, an exploration of whether and how H_2_S beneficially modifies the gut microbial profile and its effects on metabolism will provide supports for the therapeutic application of exogenous H_2_S and probiotics on type 2 diabetes metabolism. To this end, we aimed to investigate the potential relationship between the regulation of obesity as well as glucose homeostasis by exogenous H_2_S and the alterations of gut microbiota in obese db/db mice.

## Materials and Methods

### Experimental Animals and Treatment

Wild-type male C57BL/6 mice and leptin receptor-deficient (db/db) mice with the same background (10 weeks old) were purchased from the Animal Laboratory Centre of Nanjing University (Nanjing, China). The animals were housed in a climate- and temperature-controlled room, on a 12-h light/dark cycle. The mice were maintained on a standard diet and water ad libitum.

*In vivo*, H_2_S exists in two forms: 1/3 in the form of gas H_2_S and 2/3 in the form of sodium hydrosulfide (NaHS), with a dynamic equilibrium between H_2_S and NaHS (Hosoki et al., 1997). Therefore, NaHS is often used as a donor for exogenous H_2_S in experiments (Sun et al., 2018). The db/db mice treated with NaHS (80 μmol/kg; Sigma, St. Louis, MO, USA) by intraperitoneal injection every 2 days for 16 weeks (n = 6) or with equal amounts of saline (n = 6) were allocated to DB-H_2_S or DB group, respectively. Wild-type mice treated with saline were used as WT group (n = 6) (Sun et al., 2018).

All mice were visually inspected every day, and the body weight was recorded once a week. At the end of the treatment, all mice were euthanized *via* diethyl ether-induced anesthesia. The mouse serum and cecal contents were collected for the analyses of biochemical parameters and gut microbiota, respectively. Usually, in mice, the major fermentation capacity in cecum may impact on the diversity and composition of gut microbial communities that are responsible not only for the fermentation of indigestible food components but also for the production of essential complements to the host, such as vitamin K and B and short-chain fatty acids (Nguyen et al., 2015). Therefore, cecal contents are usually used in mouse gut microbiome studies (with the exception of longitudinal studies, where pellets are sampled) (Everard et al., 2014; Lambert et al., 2015; Lin et al., 2018).

The animal experiments were performed according to the Guide for the Care and Use of Laboratory Animals published by the China National Institutes of Health and approved by the Animal Care Committees of Harbin Medical University, China.

### Random Blood Glucose and Oral Glucose Tolerance Test

We randomly measured the blood glucose levels of blood samples obtained from the tail every week to verify the development of diabetes. The mice were fasted for 12 h and were administered with 50% glucose solution (2 g/kg) by gavage for the oral glucose tolerance test (OGTT). Tail vein blood was collected at 0, 15, 30, 60, 90, and 120 min after the administration. All blood samples were tested using a glucometer (Bayer, Leverkusen, Germany) (Ma et al., 2017).

### Biochemical Analyses

The measurement of H_2_S production in serum followed the established protocol (Kang et al., 2009). Briefly, serum was mixed with 10% trichloroacetic acid. The reaction was stopped by 1% zinc acetate, followed by incubation with *N*,*N*-dimethyl-p-phenylenediamine sulfate (DPD) for 15 min. The absorbance at 670 nm was measured with a spectrophotometer (Thermo Fisher Scientific, Waltham, MA, USA).

Then serum triglyceride was determined by using the mouse triglyceride (TG) ELISA Kit (Mlbio, Shanghai, China) and serum insulin was measured with the mouse insulin (INS) ELISA kit (Mlbio, Shanghai, China) according to the manufacturers’ instructions.

### DNA Isolation and Sequencing of Bacterial 16S rRNA and Fungal Internal Transcribed Spacer (ITS) Gene

Total DNA of cecal contents was extracted by using the FastDNA SPIN Extraction Kit (MP Biomedicals, Santa Ana, CA, USA). The quantity and quality of extracted DNA were measured using a spectrophotometer (Thermo Fisher Scientific, Waltham, MA, USA) and agarose gel electrophoresis, respectively.

The V3–V4 regions of the bacterial 16S rRNA genes were amplified using primers 338F (5′-ACTCCTACGGGAGGCAGCA-3′) and 806R (5′-GGACTACHVGGGTWTCTAAT-3′). The internal transcribed spacer (ITS) regions of fungi were amplified with primers ITS5F (5′-GGAAGTAAAAGTCGTAACAAGG-3′) and ITS1R (5′-GCTGCGTTCTTCATCGATGC-3′). Sample-specific 7-bp barcodes were incorporated into the primers for multiplex sequencing. The detailed PCR components and procedures were conducted according to a previous method (Zhou et al., 2019). PCR amplicons were purified using Agencourt AMPure Beads (Beckman Coulter, Indianapolis, IN, USA) and quantified with the PicoGreen dsDNA Assay Kit (Invitrogen, Carlsbad, CA, USA). After the individual quantification step, amplicons were pooled in equal amounts and subjected to paired-end 2 × 300-bp sequencing using the Illumina MiSeq platform and the MiSeq Reagent Kit v3 from Shanghai Personal Biotechnology Co., Ltd. (Shanghai, China).

### Sequencing Data Processing and Analyses

Sequencing data were processed using a quantitative analysis of microbial ecology (QIIME, v1.8.0). Briefly, raw sequencing reads that exactly matched the barcode were assigned to the corresponding samples and identified as valid sequences. The low-quality sequences (length < 150 bp, average Phred scores < 20, containing ambiguous bases, and single-nucleotide repeats > 8 bp) were filtered (Gill et al., 2006; Chen and Jiang, 2014). Paired-end reads were assembled using FLASH (Magoc and Salzberg, 2011). After chimera detection, the remaining high-quality sequences were clustered into operational taxonomic units (OTUs) at 97% sequence identity by UCLUST (Edgar, 2010). The default parameters were used to select the representative sequence from each OTU. OTU taxonomy classification was performed by BLAST searching the representative set of sequences against the Greengenes database (DeSantis et al., 2006). An OTU table was further generated to record the abundance of each OTU in each sample and the taxonomy of these OTUs. OTUs with a total content of less than 0.001% in all samples were discarded (Bokulich et al., 2013).

Sequencing data analyses were mainly performed using QIIME (version 1.8.0) and R packages (version 3.2.0). OTU-level alpha-diversity indices including Chao1 and Shannon were calculated using the OTU table in QIIME. Rarefaction plots were generated with iterations of 20 at each sampling depth of 10 and increments of 500. The unweighted and weighted UniFrac distance matrices were calculated and used for principal coordinate analysis (PCoA), and analysis of similarities (ANOSIM) was processed in QIIME. Bubble charts containing bacterial genus taxa with a relative abundance ≥ 0.1% and fungal genus taxa with a relative abundance ≥ 0.5% in at least one group were created by R software.

The sequences generated in this study are available in the National Center for Biotechnology Information (NCBI) Sequence Read Archive (accession number PRJNA780813).

### Statistical Analyses

Statistical analyses and graphing were performed using GraphPad Prism software (version 6.0). The area under the curve (AUC) for each OGTT was calculated through trapezoidal approximation. All data are presented as mean ± standard error of mean (SEM). Differences among groups for statistical significance were determined using one-way analysis of variance (ANOVA) followed by Tukey’s multiple-comparison test or Kruskal–Wallis test followed by Dunn’s multiple-comparison test. Correlations between bacterial or fungal abundance and metabolic parameters were assessed by Spearman’s correlation analysis. A *p*-value < 0.05 was considered statistically significant.

## Results

### Effects of Exogenous H_2_S on Serum H_2_S Level, Obesity Parameters and Glucose Homeostasis

Previous studies have reported the increase of body weight, glucose intolerance, and the levels of serum glucose, serum insulin, serum triglyceride, and free fatty acid in the db/db mice, recapitulating hallmark features of type 2 diabetes (Ma et al., 2017; Sun et al., 2018; Sun et al., 2019). By treating with NaHS for 10 or 12 weeks, db/db mice displayed improvements in glucose tolerance and serum insulin levels, while treatment for 12 weeks also decreased the plasma free fatty acid levels in db/db mice (Sun et al., 2018; Sun et al., 2019).

**Figure 1:**
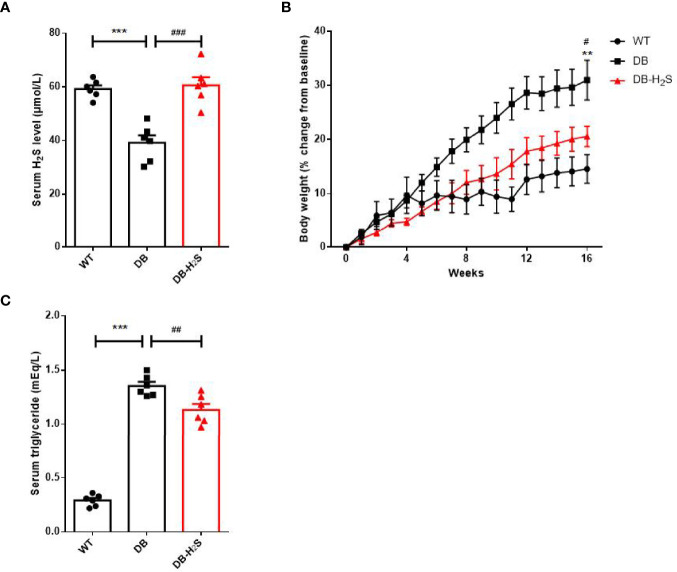
Effects of exogenous H_2_S on serum H_2_S level and obesity parameters. **(A)** Serum H_2_S level at week 16. **(B)** Percent change in body weight from baseline over 16 weeks. **(C)** Serum triglyceride level at week 16. WT, wild-type mouse group (n = 6); DB, db/db mouse group (n = 6); and DB-H_2_S, db/db mouse treated with NaHS group (n = 6). Data are shown as mean ± SEM. Differences were analyzed by one-way ANOVA with Tukey’s multiple-comparison test and denoted as follows: **p < 0.01, ***p < 0.001, WT vs. DB; ^#^ *p* < 0.05, ^##^ *p* < 0.01, ^###^ *p* < 0.001, DB-H_2_S vs. DB.

Here, we measured the body weight, serum H_2_S, triglyceride, and glucose levels to explore whether a longer time of NaHS treatment could prevent the obesity in db/db mice (**Figure 1**). We found that the db/db mice had a significant decrease in serum H_2_S level compared with wild-type mice. By treatment with NaHS for 16 weeks, the mice already showed obvious improvement in serum H_2_S level compared to db/db mice (**Figure 1A**). NaHS treatment significantly improved the obesity condition by slowing the body weight gain (**Figure 1B** and **Supplementary Figure 1**), decreasing the level of serum triglyceride in db/db mice (**Figure 1C**).

**Figure 2:**
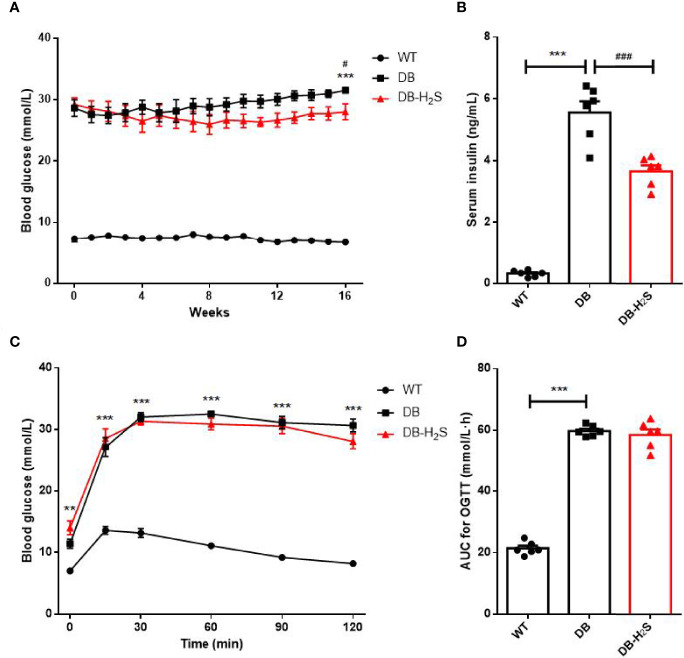
Effects of exogenous H_2_S on glucose homeostasis. **(A)** Random blood glucose. **(B)** Serum insulin level at week 16. **(C)** Oral glucose tolerance test (OGTT) at week 16. **(D)** Area under the curve (AUC) for the OGTT. WT, wild-type mouse group (n = 6); DB, db/db mouse group (n = 6); DB-H_2_S, db/db mouse treated with NaHS group (n = 6). Data are shown as mean ± SEM. Differences were analyzed by one-way ANOVA with Tukey’s multiple-comparison test and denoted as follows: **p < 0.01, ***p < 0.001, WT vs. DB; ^#^ *p* < 0.05, ^###^ *p <*0.001, DB-H_2_S vs. DB.

Furthermore, administration of NaHS for 16 weeks induced a modest but significant decrease in the blood glucose level of db/db mice (**Figure 2A**). Similarly, a significantly lower level of serum insulin (**Figure 2B**) occurred in the treated db/db mice at the end of study. The results of OGTT at week 16 are presented in **Figures 2C, D**. The blood glucose baselines for OGTT started at relatively high levels in DB and DB-H_2_S groups, and glucose tolerance in the DB-H_2_S group was not obviously improved compared to the DB group after glucose administration. Likewise, the DB-H_2_S group did not have a significantly lower AUC value for OGTT compared to the DB group. These results suggested that NaHS administration was able to alleviate hyperglycemia but difficult to ameliorate impaired glucose tolerance.

### Effect of Exogenous H_2_S on the Gut Bacterial Microbiota Community Structure

It has been reported that compared to wild-type mice, db/db mice show a significantly different gut microbiota composition, especially the increased level of the phylum Firmicutes and decreased level of the phylum Bacteroidetes (Zheng et al., 2018). To further determine the relationship between exogenous H_2_S, obesity condition, glucose metabolism, and the gut microbiota, we analyzed the gut microbiota of db/db mice after treatment with NaHS for 16 weeks.

Firstly, the gut bacterial microbiota was analyzed by sequencing the V3–V4 regions of the bacterial 16S rRNA gene. After removing the low-quality sequences, an average 30,772 (20,126–44,585) clean reads were generated from each sample. The high-quality sequences were then delineated into 3,779 OTUs (243–1,605 OTUs per sample) on the basis of 97% similarity. The sample tags and OTUs are shown in **Supplementary Table 1**. The observed species and Shannon diversity rarefaction curves reached the saturation phase. This indicated that the sequence depth obtained was adequate for all samples (**Supplementary Figure 2**).

**Figure 3:**
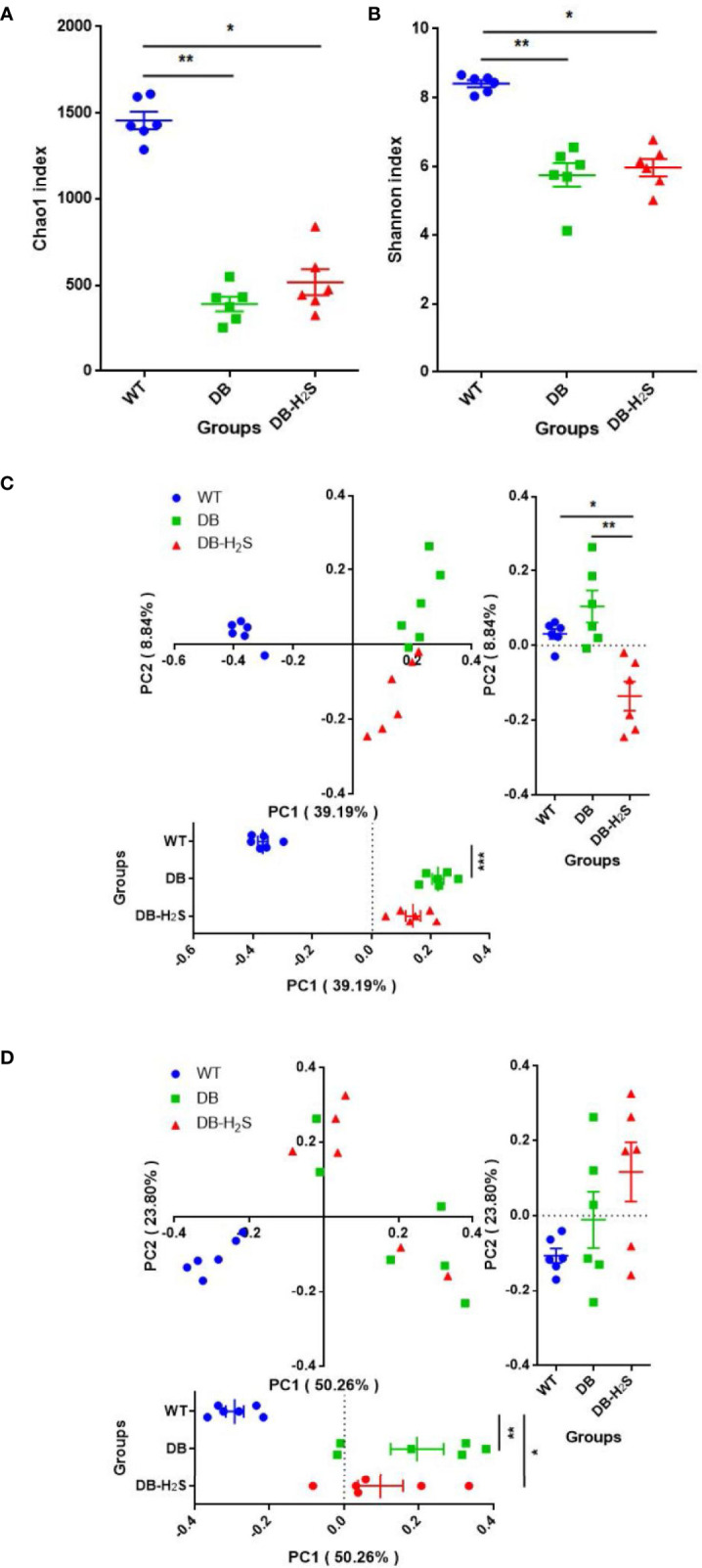
Effect of exogenous H_2_S on the gut microbiota structure. **(A)** Chao1 index. **(B)** Shannon index. **(C)** Unweighted UniFrac distance-based principal coordinate analysis (PCoA). **(D)** Weighted UniFrac distance-based PCoA. WT, wild-type mouse group (n = 6); DB, db/db mouse group (n = 6); DB-H_2_S, db/db mouse treated with NaHS group (n = 6). Data are shown as mean ± SEM. Differences were analyzed by the Kruskal–Wallis test with Dunn’s multiple-comparison test and denoted as follows: *p < 0.05, **p < 0.01, ***p < 0.001.

We analyzed the alpha-diversity, which consisted of community richness and diversity (richness and evenness), among the three groups (**Figures 3A, B**). The bacterial richness (represented by the Chao1 index) and diversity (represented by the Shannon index) in the WT group were significantly higher than those in DB and DB-H_2_S groups (**Figures 3A, B**). However, exogenous H_2_S failed to alter the alpha-diversity indicating that this treatment was not beneficial for the recovery of the richness and diversity of the microbial community in db/db mice.

Beta-diversity analysis was performed based on the unweighted and weighted UniFrac distance-based PCoA (**Figures 3C, D**). Unweighted UniFrac metrics clearly showed different microbial structures in the three groups (ANOSIM, *R* = 0.7486, *p* = 0.001) (**Figure 3C**). Compared to the DB group, the DB-H_2_S group had a more similar gut bacterial microbiota structure with that in the WT group at the first principal coordinate (PC1) (**Figure 3C**). The PCoA, based on weighted UniFrac metrics, shows distinct clustering (ANOSIM, *R* = 0.5819, *p* = 0.001) of groups by diabetes rather than treatment (**Figure 3D**). Notably, the bacterial community structure among the mice in the WT group was very similar, while that in the mice of the DB group as well as DB-H_2_S had apparent heterogeneity (**Figures 3C, D**). These results indicated that the exogenous H_2_S intervention may have benefits on gut bacterial microbiota structure shift to that in the mice of the WT group.

### Effect of Exogenous H_2_S on the Gut Bacterial Microbiota Composition

**Figure 4:**
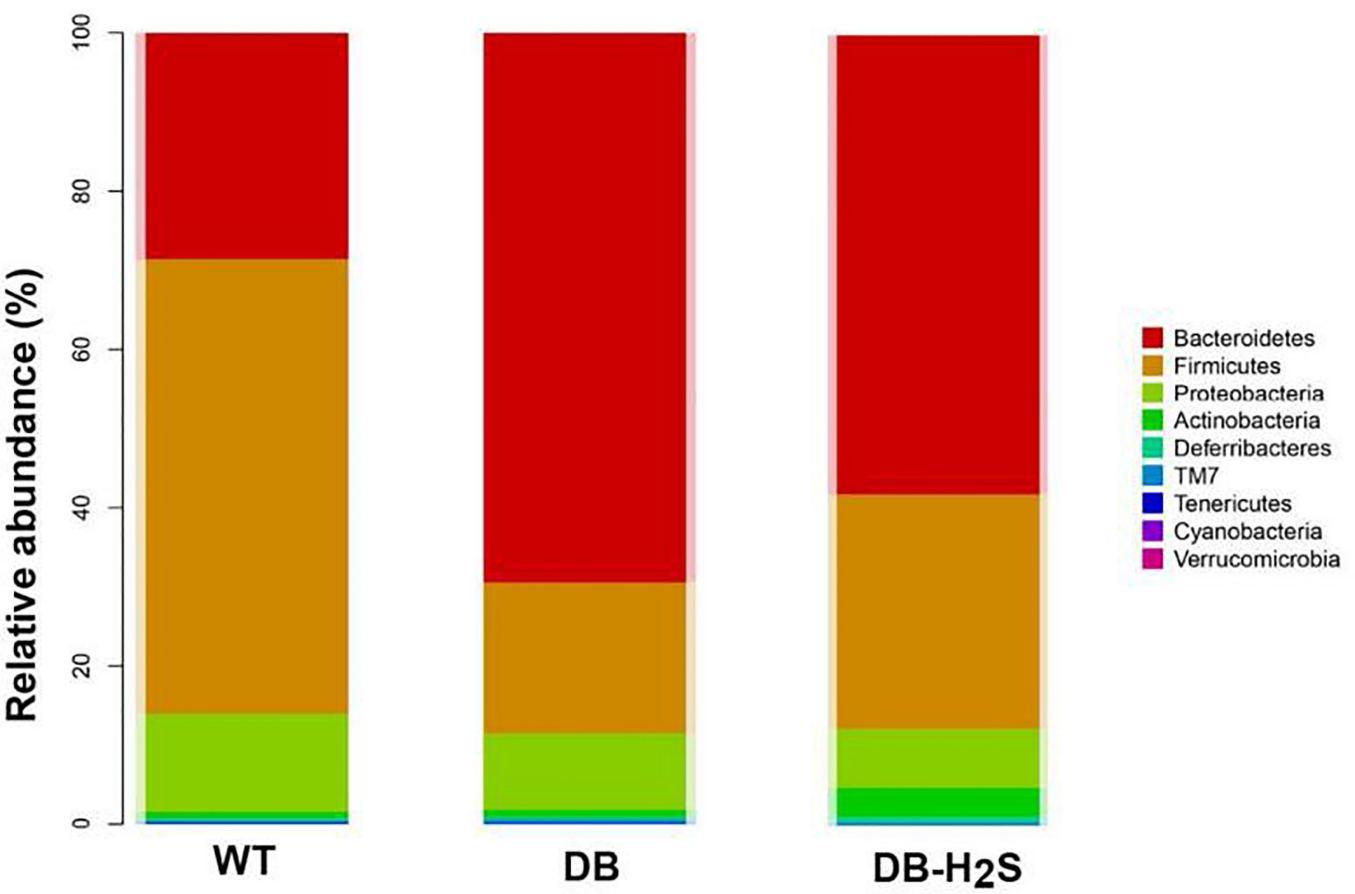
Effect of exogenous H_2_S on the gut microbiota composition. Changes in bacterial taxonomic composition of the gut microbiota at the phylum level. WT, wild-type mouse group (n = 6); DB, db/db mouse group (n = 6); DB-H_2_S, db/db mouse treated with NaHS group (n = 6).

To further understand the microbial composition among the mice of the three groups, taxonomy-based analysis at the phylum and genus levels was performed (**Figure 4** and **Supplementary Figure 3**). We found that more than 99% of the sequences were within the top three phyla, Firmicutes, Bacteroidetes, and Proteobacteria (**Figure 4**). Compared with the WT group, the decreased level of Firmicutes (57.43% versus 19.07%) and increased level of Bacteroidetes (28.58% versus 69.45%) were observed in the DB group. NaHS treatment increased the relative abundance of Firmicutes (29.64% versus 19.07%) and suppressed Bacteroidetes (58.15% versus 69.45%) in db/db mice (**Figure 4**).

At the genus level, the 20 most abundant genera displayed all changes among the different groups (**Supplementary Table 2**). As shown in **Table 1** and **Supplementary Figure 3**, the bacterial composition at the genus level tended to be dispersed. The relative abundance of genera like *Unclassified_S24-7*, *Bacteroides*, *Lactobacillus*, *Unclassified_Enterobacteriaceae*, [*Prevotella*], and *Prevotella* in the WT group decreased compared with the DB group. Meanwhile, *Unclassified_Ruminococcaceae*, *Helicobacter*, *Oscillospira*, *Desulfovibrio*, [*Ruminococcus*], *Ruminococcus*, and *Sutterella* increased compared with the DB group. By treatment with exogenous H_2_S, the db/db mice already showed an improvement in the above high-abundance bacterial genera, such as lessened *Unclassified_S24-7* and enhancive *Unclassified_Ruminococcaceae*.

**Table 1:** The relative abundance of bacterial genera that showed an improvement by treatment with exogenous NaHS.

Phylum	Genus	WT	DB	DB-H_2_S	*p*-value*^a^*
Bacteroidetes	*Unclassified_S24-7*	25.42% ± 0.0257	51.85% ± 0.0929	42.62% ± 0.0286	0.0178
	*Bacteroides*	0.13% ± 0.0003	5.40% ± 0.0269	4.23% ± 0.0131	0.1110
	*Prevotella*	0.08% ± 0.0004	2.19% ± 0.0099	1.14% ± 0.0082	0.1739
	*[Prevotella]*	1.44% ± 0.0046	2.06% ± 0.0064	1.01% ± 0.0054	0.4289
Firmicutes	*Lactobacillus*	0.59% ± 0.0038	4.11% ± 0.0143	3.02% ± 0.0132	0.1207
	*Ruminococcus*	0.96% ± 0.0025	0.42% ± 0.0012	0.55% ± 0.0021	0.1815
	*Oscillospira*	2.79% ± 0.0045	0.45% ± 0.0008	0.66% ± 0.0020	< 0.0001
	*Unclassified_Peptostreptococcaceae*	0.09% ± 0.0004	0.93% ± 0.0058	0.24% ± 0.0008	0.2049
	*Unclassified_Lachnospiraceae*	7.08% ± 0.0129	0.89% ± 0.0026	1.19% ± 0.0048	< 0.0001
	*Unclassified_Ruminococcaceae*	2.50% ± 0.0037	0.88% ± 0.0026	4.87% ± 0.0345	0.3934
	*[Ruminococcus]*	1.81% ± 0.0050	0.72% ± 0.0029	1.22% ± 0.0084	0.4513
Proteobacteria	*Helicobacter*	3.84% ± 0.0246	0.58% ± 0.0040	1.77% ± 0.0066	0.3227
	*Unclassified_Enterobacteriaceae*	0.01% ± 0.0001	4.71% ± 0.0286	2.35% ± 0.0198	0.2835
	*Desulfovibrio*	2.45% ± 0.0101	0.31% ± 0.0009	0.99% ± 0.0058	0.1061
	*Sutterella*	0.76% ± 0.0025	0.18% ± 0.0009	0.53% ± 0.0016	0.1137

a Data are presented as mean ± SEM and analyzed by one-way ANOVA.

### Effect of Exogenous H_2_S on the Gut Mycobiome

Next, the gut mycobiome was analyzed by sequencing the ITS region of the fungal gene to further explore whether exogenous H_2_S may alter the gut mycobiome. In fact, the mycobiome, referring principally to the fungal component of microbiota, comprises approximately 0.03%–2% of total gut microorganisms (Mar Rodriguez et al., 2015). Therefore, analyzing mycobiome may provide additional information of gut microbiota of db/db mice and exogenous H_2_S treatment effects.

**Figure 5:**
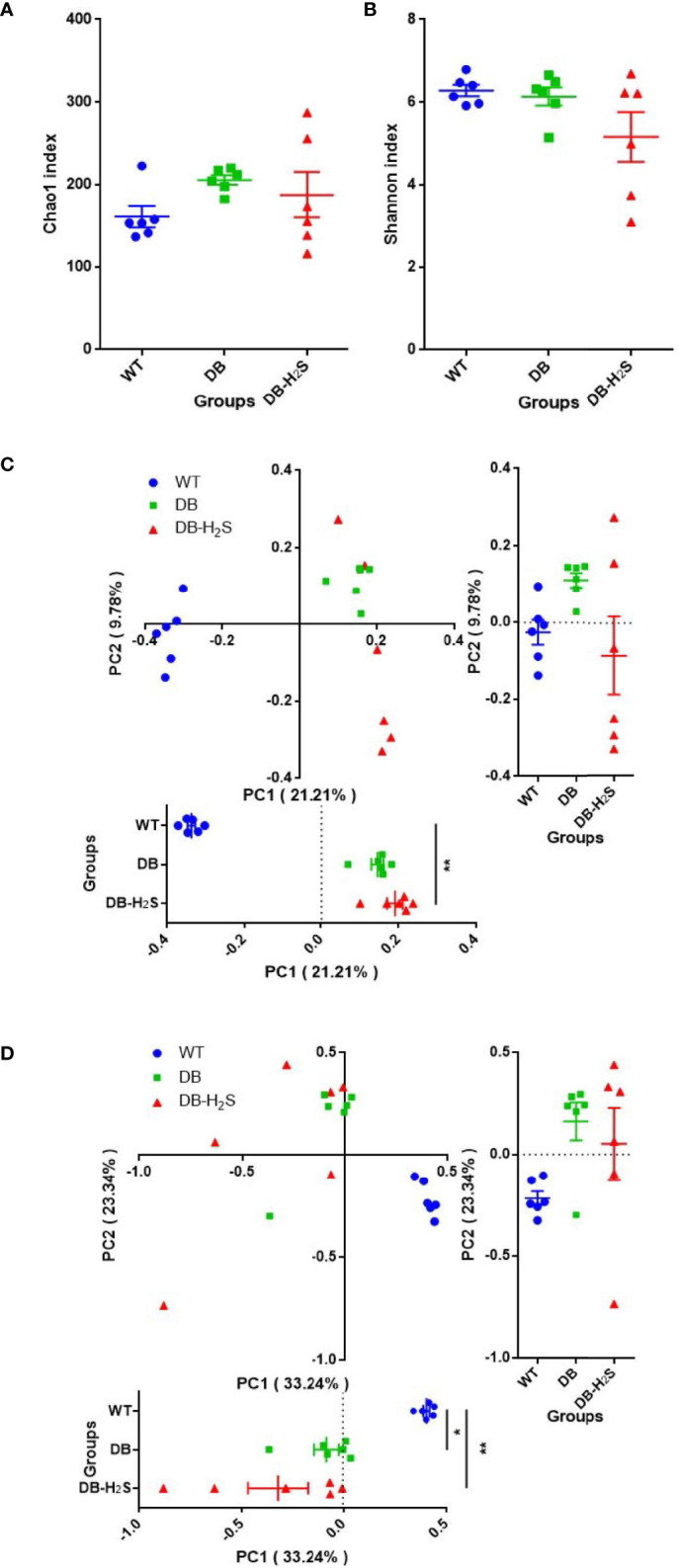
Effect of exogenous H_2_S on the gut mycobiome structure. **(A)** Chao1 index. **(B)** Shannon index. **(C)** Unweighted UniFrac distance-based principal coordinate analysis (PCoA). **(D)** Weighted UniFrac distance-based PCoA. WT, wild-type mouse group (n = 6); DB, db/db mouse group (n = 6); DB-H_2_S, db/db mouse treated with NaHS group (n = 6). Data are shown as mean ± SEM. Differences were analyzed by the Kruskal–Wallis test with Dunn’s multiple-comparison test and denoted as follows: *p < 0.05, **p < 0.01.

Sequencing effort yielded 778,583 sequence reads (range 27,870−62,386), which were binned into 1,415 OTUs. The fungal observed species and Shannon diversity rarefaction curves also reached the saturation phase (**Supplementary Figure 4**). There were no significant differences in the richness and diversity of fungal species in the mice of all groups (**Figures 5A, B**). Fungal structures by PCoA were markedly different among the three groups (unweighted, ANOSIM, *R* = 0.7885, *p* = 0.001; weighted, ANOSIM, *R* = 0.4683, *p* = 0.001), while the DB-H_2_S group revealed a more dispersed cluster (**Figures 5C, D**).

**Figure 6:**
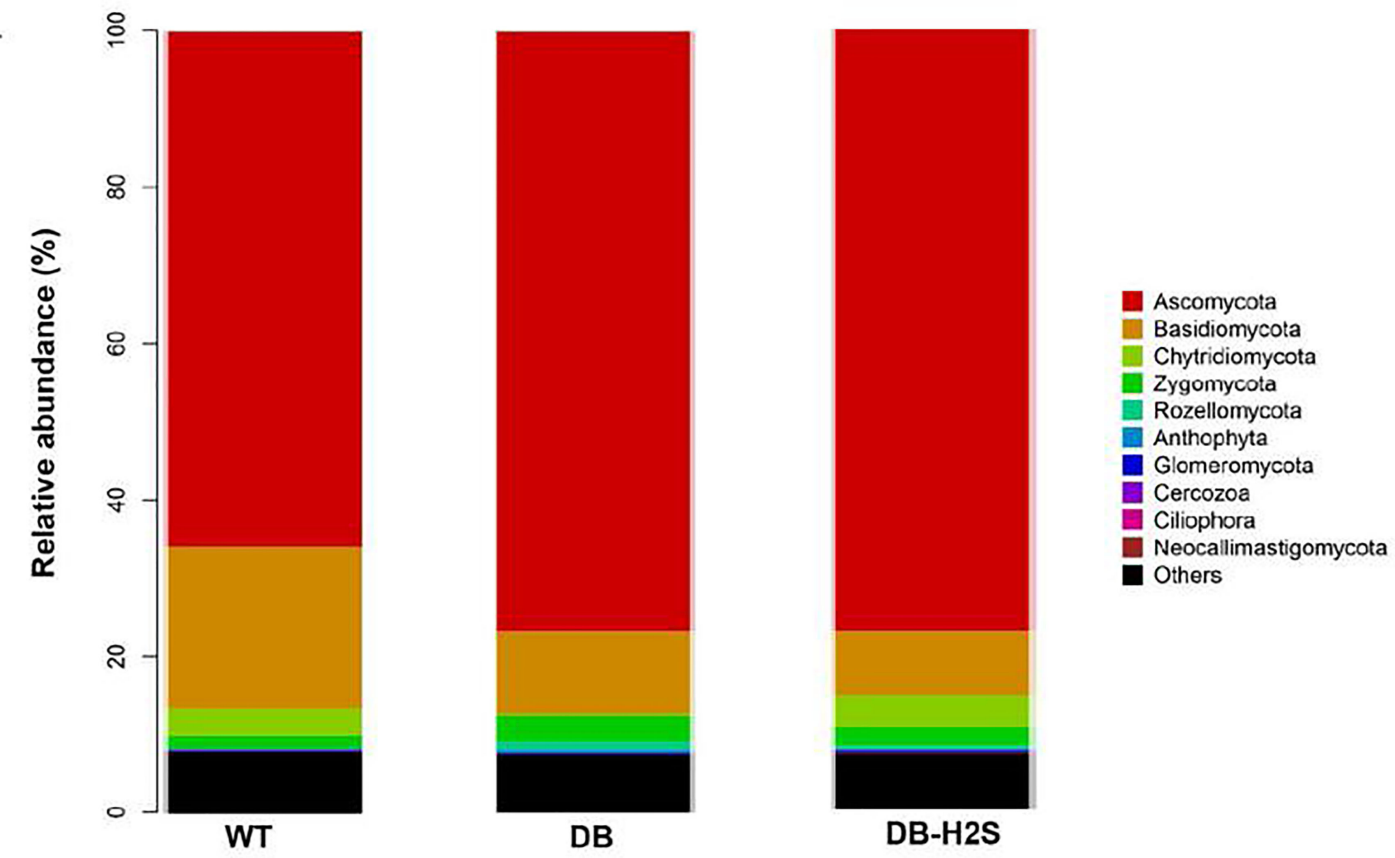
Effect of exogenous H_2_S on the gut mycobiome composition. Changes in the fungal taxonomic composition of the gut microbiota at the phylum level. WT, wild-type mouse group (n = 6); DB, db/db mouse group (n = 6); DB-H_2_S, db/db mouse treated with NaHS group (n = 6).

At the phylum level, 80%–90% of the gut fungal community (in the cecum content) was dominated by Ascomycota and Basidiomycota (**Figure 6**). Bubble charts exhibited that in the WT group, *Candida*, *Aspergillus*, *Trichosporon*, and *Mortierella* reduced in the genus level compared with the DB group. Compared to the DB group, the relative abundance of genera like *Candida*, *Aspergillus*, *Trichosporon*, and *Mortierella* also has a decline in the DB-H_2_S group (**Table 2** and **Supplementary Figure 5**). Furthermore, we found that the *Simplicillium* and *Parmelina* genera were enriched in the DB-H_2_S group (**Supplementary Figure 5**).

**Table 2:** The relative abundance of fungal genera that showed an improvement by treatment with exogenous NaHS.

Phylum	Genus	WT	DB	DB-H_2_S	*p*-value*^a^*
Ascomycota	*Aspergillus*	0.96% ± 0.0033	3.31% ± 0.0079	1.90% ± 0.0062	0.0478
	*Candida*	0.63% ± 0.0022	5.33% ± 0.0318	0.22% ± 0.0014	0.1273
	*Fusarium*	0.92% ± 0.0025	0.07% ± 0.0006	0.31% ± 0.0016	0.0122
	*Zopfiella*	0.70% ± 0.0036	1.26% ± 0.0038	0.81% ± 0.0035	0.5357
	*Archaeorhizomyces*	0.20% ± 0.0009	1.85% ± 0.0147	0.43% ± 0.0019	0.3655
	*Staphylotrichum*	0.20% ± 0.0011	0.97% ± 0.0032	0.20% ± 0.0012	0.0295
	*Acremonium*	6.65% ± 0.0042	0.91% ± 0.0028	2.23% ± 0.0109	< 0.0001
	*Myrothecium*	0.63% ± 0.0017	0.01% ± 0.0001	0.13% ± 0.0013	0.0083
Basidiomycota	*Trichosporon*	0.09% ± 0.0009	0.55% ± 0.0035	0.10% ± 0.0005	0.2545
Zygomycota	*Mortierella*	1.05% ± 0.0042	1.98% ± 0.0075	0.86% ± 0.0040	0.3299

a Data are presented as mean ± SEM and analyzed by one-way ANOVA.

### Correlations Between Metabolic Parameters and Exogenous H_2_S-Induced Alterations of the Gut Microbiota

**Figure 7:**
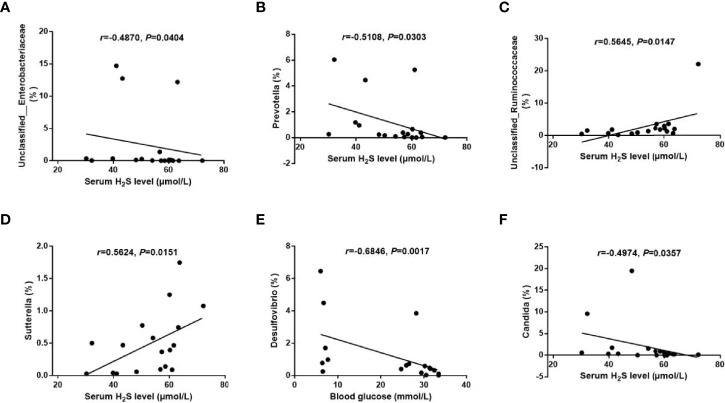
Correlations between the metabolic parameters and the gut microbiota abundance. **(A–D)**: Correlations of the serum H_2_S level with the abundance of bacterial genera *Unclassified_Enterobacteriaceae* **(A)**, *Prevotella* **(B)**, *Unclassified_Ruminococcaceae* **(C)**, and *Sutterella* **(D)**. **(E)** Correlation of the blood glucose level with the abundance of bacterial genus *Desulfovibrio*. **(F)** Correlation of the serum H_2_S level with the abundance of fungal genus *Candida.* The data were analyzed by Spearman correlation analysis.

Spearman correlation analysis was performed to determine the correlations between the metabolic parameters and the microbial abundance at the genus level. The analysis revealed significant negative correlations between the serum H_2_S level and the abundances of *Unclassified_Enterobacteriaceae* (*r* = −0.4870, *p* = 0.0404) and *Prevotella* (*r* = −0.5108, *p* = 0.0303), and positive correlations between the serum H_2_S level and the abundances of *Unclassified_Ruminococcaceae* (*r* = 0.5645, *p* = 0.0147) and *Sutterella* (*r* = 0.5624, *p* = 0.0151) (**Figures 7A**–**D**). Meanwhile, we found a significant negative correlation between the blood glucose level and the abundance of *Desulfovibrio* (*r* = −0.6846, *p* = 0.0017) (**Figure 7E**). In addition, a significant negative correlation of the serum H_2_S level with the abundance of fungal genus *Candida* was discovered (*r* = −0.4974, *p* = 0.0357) (**Figure 7F**).

## Discussion

This study demonstrated that NaHS administration in db/db mice modifies host metabolism and is associated with changes in the gut microbial composition. NaHS-treated mice exhibited a reduced body weight gain ratio, serum triglyceride, blood glucose, and serum insulin, thereby suggesting that the NaHS treatment manner in this study may also act as a beneficial function in the context of obesity and type 2 diabetes. To our knowledge, this is a new high-throughput study analyzing the effects of exogenous H_2_S on the gut microbiota and the first study showing the pattern of gut fungal mycobiome in db/db mice.

Except that H_2_S formation in the pancreas and liver was increased in diabetic rats (Yusuf et al., 2005), decreased H_2_S level in blood in patients or various animal models of diabetes has been mostly reported (Brancaleone et al., 2008; Jain et al., 2010; Peake et al., 2013; Gheibi et al., 2019). In this study, NaHS administration increased serum H_2_S in db/db mice and reached the serum H_2_S level in wild-type mice. Recently, Gheibi et al. have explained the reasons for the lower blood H_2_S levels: hyperglycemia results in an increase in H_2_S consumption and the activities of H_2_S-generating enzymes are lower in blood of diabetic patients (Gheibi et al., 2019). These are consistent with our results reported in this study. The activity and expression of H_2_S-synthesizing enzymes were increased in the pancreas and liver which also result in a higher generation amount of H_2_S (Yusuf et al., 2005). In terms of glucose homeostasis, exogenous H_2_S significantly decreased blood glucose but did not improve glucose tolerance in our present study. A thorough and longitudinal treatment protocol should be designed to demonstrate the controversy about the role of H_2_S in diabetes and find effective therapeutic strategies by regulating the H_2_S level.

Here, we found that gut-dominating bacterial phyla identified in this study were in line with the findings of other studies (Song et al., 2017; Zheng et al., 2018). The changes in the relative abundance of the two dominant bacterial divisions, Firmicutes and Bacteroidetes, have been widely reported to associate with obesity (Ley et al., 2005; Turnbaugh et al., 2006) or type 2 diabetes (Everard et al., 2011; Geurts et al., 2011). In this regard, the increased ratio of Firmicutes to Bacteroidetes was generally considered as a marker of gut dysbiosis in obesity and type 2 diabetes. However, these in some literature reports were not uniformly observed (Duncan et al., 2008; Larsen et al., 2010). Also, dietary capsaicin has been reported to improve glucose homeostasis with an increase in the phylum Firmicutes and a corresponding decrease in the Bacteroidetes in obese diabetic ob/ob mice (Song et al., 2017) which is similar to exogenous H_2_S reported here. Therefore, the ratio of Firmicutes to Bacteroidetes might only be an indicator, but a detailed view at the genus level is even more important in regard to distinction of their functions.

In this study, we observed that *Unclassified_Enterobacteriaceae* and *Prevotella* decreased after NaHS administration and negatively correlated with the serum H_2_S level. The population of Enterobacteriaceae, a family containing several opportunistic pathogens, has been reported to induce obesity and insulin resistance (Fei and Zhao, 2013). The species of *Prevotella* was also demonstrated to induce insulin resistance (Pedersen et al., 2016). Moreover, the Lachnospiraceae (including [*Ruminococcus*] genus here) and Ruminococcaceae families (including *Unclassified_Ruminococcaceae*, *Oscillospira*, and *Ruminococcus* genera here) were two main butyrate-producing taxonomic groups and showed to be associated with healthier phenotypes (Garcia-Mazcorro et al., 2016). Moreover, our results demonstrated that these bacteria were increased in NaHS-treated db/db mice. Interestingly, *Lactobacillus* with probiotic characteristics was found to have higher abundance in db/db mice in our study. Increased abundance of *Lactobacillus* was also observed in long-standing diabetic subjects (Bhute et al., 2017). This may be related to different species of the genus *Lactobacillus*. Some earlier studies have suggested that germ-free mice have between 50% and 80% less H_2_S in their tissues and circulation (Shen et al., 2013). The gut microbiota break down protein and complex carbohydrates into short-chain fatty acids and gases (e.g., hydrogen) that are utilized by sulfate-reducing bacteria to produce H_2_S. In a study of healthy individuals in the United States, approximately 50% of those had their gut colonized by sulfate-reducing bacteria, with a member of the genus of *Desulfovibrio* being the primary H_2_S producer (Rey et al., 2013). In this study, we observed that *Desulfovibrio* increased after NaHS administration, although the difference was not statistically significant. Previous studies also have shown that there was no significant difference in abundance of *Desulfovibrio* between the wild-type mice and treated mice (Hsu et al., 2021). Since *Desulfovibrio* was proposed as an inflammation activator, the effects of exogenous and endogenous H_2_S on gut inflammation should be investigated extensively. Currently, the role of H_2_S in intestinal inflammation is complex and sometimes contradictory. The therapeutic delivery of exogenous H_2_S into the gut restored the microbiota biofilm and mucus production and reduced gut inflammation (Motta et al., 2015). Thus, we believe that the beneficial effects of exogenous H_2_S on type 2 diabetes may be induced by metabolites associated with the specific bacterial changes at the genus level. The association needs to be further validated by metabolomics.

There are a few studies about the mycobiome of the type 2 diabetes population in comparison to healthy controls. For instance, a pilot study has suggested that *candida* appears to be more prevalent in the feces of patients with type 2 diabetes (Gosiewski et al., 2014). A research about Indian type 2 diabetic subjects has shown that opportunistic fungal pathogens such as *Candida* and *Aspergillus* were found to be enriched in newly diagnosed diabetic subjects (Bhute et al., 2017). To our knowledge, no study exists about the gut mycobiome of diabetic animal models. Next-generation sequencing will be valuable for characterizing the gut mycobiome associated with metabolism disorder. Here, that the fungal structure in db/db mice has changed substantially reflects the close association of gut mycobiome with diabetes. Consequently, it is speculated that gut dysbiosis in diabetes creates the environment for fungal overgrowth. We also observed that *Candida* and *Aspergillus* decreased after NaHS administration and *Candida* negatively correlated with the serum H_2_S level. Although for decades fungi are considered harmful to their host, the modifications of fungal communities in the gut should be paid more attention.

In conclusion, we found that exogenous H_2_S led to significant improvement of diabetic metabolic disorders in db/db mice. The beneficial effects of exogenous H_2_S on diabetic metabolic disorders are likely related to the alterations of both bacterial and fungal microbiota. Some remarkable genera were proved to possess significant correlations with the serum H_2_S and blood glucose levels. This relationship can still be found in the gut mycobiome. These results offer a novel insight that alterations in the gut microbiota composition may be the potential mechanism underlying the effects of exogenous H_2_S on diabetic metabolic disorders. The main strength of this research is that a role of exogenous H_2_S intervention in type 2 diabetes was associated with gut microbiota (bacterial and fungal aspects). This study has some limitations, in that our results were based on a relatively small sample size and the cross-sectional design. Lastly, we have not demonstrated a causal relationship between gut microbiota and exogenous H_2_S-mediated metabolic improvement in db/db mice.

## Data Availability Statement

The original contributions presented in the study are publicly available. These data can be found as follows: NCBI, PRJNA780813.

## Ethics Statement

The animal study was reviewed and approved by the Animal Care Committees of Harbin Medical University, China.

## Author Contributions

HL and W-HZ conducted the study and designed the experiment. LL and WZ performed the experiments. JL and Z-WG performed the data analysis. JL and Z-WG wrote the draft of the manuscript and revised the manuscript. All authors contributed to the article and approved the submitted version.

## Funding

This work was supported by the Graduate Innovation Foundation of Harbin Medical University (YJSCX2016-41HYD) and National Science Foundation of China (81970317).

## Conflict of Interest

The authors declare that the research was conducted in the absence of any commercial or financial relationships that could be construed as a potential conflict of interest.

## Publisher’s Note

All claims expressed in this article are solely those of the authors and do not necessarily represent those of their affiliated organizations, or those of the publisher, the editors and the reviewers. Any product that may be evaluated in this article, or claim that may be made by its manufacturer, is not guaranteed or endorsed by the publisher.
