# Supplementary material for: Effects of Exogenous Hydrogen Sulfide on Diabetic Metabolic Disorders in db/db Mice Are Associated With Gut Bacterial and Fungal Microbiota
Source: Front Cell Infect Microbiol. 2022 Mar 29;12:801331. doi: 10.3389/fcimb.2022.801331 (PMC9001961; doi:10.3389/fcimb.2022.801331)
Supplement: Supplementary file 1 [file DataSheet_1.pdf]

Effects of Exogenous Hydrogen Sulfide on Diabetic Metabolic Disorders in db/db Mice are Associated with Gut Bacterial and Fungal Microbiota

Jian Liu [1†], Wei Zhao[1†], Zi-Wei Gao[1†], Ling Liu[2], Wei-Hua Zhang[2*] and Hong Ling[1,3,4,5*]

[1] Department of Microbiology, Harbin Medical University, Harbin, China, [2] Department of Pathophysiology, Harbin Medical University, Harbin, China, [3]Wu Lien-Teh Institute, Harbin Medical University, Harbin, China, [4] Heilongjiang Provincial Key Laboratory of Infection and Immunity, Harbin, China, [5] Key Laboratory of Pathogen Biology, Harbin, China

*** Correspondence:**

Hong Ling, Harbin Medical University, Harbin, 150081, Heilongjiang, China

Weihua Zhang, Harbin Medical University, Harbin, 150081, Heilongjiang, China

Table S1 | Summary of the sample tags and OTUs.

Sample	Clean Tags	Valid Tags	Valid Percent	OTU Counts
WT1	26595	22153	83.29%	1575
WT2	25187	21270	84.44%	1397
WT3	26018	21719	83.47%	1605
WT4	24361	19676	80.76%	1426
WT5	23066	18675	80.96%	1433
WT6	21983	18548	84.37%	1289
DB1	33128	32644	98.53%	410
DB2	38369	37903	98.78%	395
DB3	34829	34450	98.91%	355
DB4	40443	39757	98.30%	288
DB5	34167	33525	98.12%	511
DB6	44585	44088	98.88%	243
DB-H_2S1	37110	36435	98.18%	393
DB-H_2S2	38074	37593	98.73%	300
DB-H_2S3	32878	32297	98.23%	525
DB-H_2S4	29524	28256	95.70%	753
DB-H_2S5	20126	19305	95.92%	477
DB-H_2S6	23453	22958	97.88%	407

Table S2 | The relative abundance of high-abundant bacterial genera in each group.

Genus	WT	DB	DB-H_2S
Unclassified_S24-7	25.43%	51.84%	42.62%
Unclassified_Clostridiales	37.89%	5.50%	4.01%
Bacteroides	0.13%	5.40%	4.23%
Unclassified_Enterobacteriaceae	0.01%	4.71%	2.34%
Lactobacillus	0.59%	4.11%	3.02%
Unclassified_Rikenellaceae	0.29%	2.87%	3.12%
Turicibacter	0.60%	2.68%	5.95%
Unclassified_Desulfovibrionaceae	5.27%	2.62%	0.33%
Prevotella	0.08%	2.19%	1.14%
[Prevotella]	1.44%	2.06%	1.01%
Odoribacter	0.23%	1.83%	1.97%
Parabacteroides	0.14%	1.77%	2.27%
Unclassified_Peptostreptococcaceae	0.09%	0.93%	0.24%
Unclassified_Lachnospiraceae	7.08%	0.89%	1.19%
Unclassified_Ruminococcaceae	2.50%	0.88%	4.87%
Unclassified_Bacteroidales	0.23%	0.85%	1.21%
[Ruminococcus]	1.81%	0.72%	1.22%
Helicobacter	3.84%	0.58%	1.77%
Unclassified_F16	0.28%	0.53%	0.36%
Unclassified_Helicobacteraceae	0.01%	0.50%	1.32%

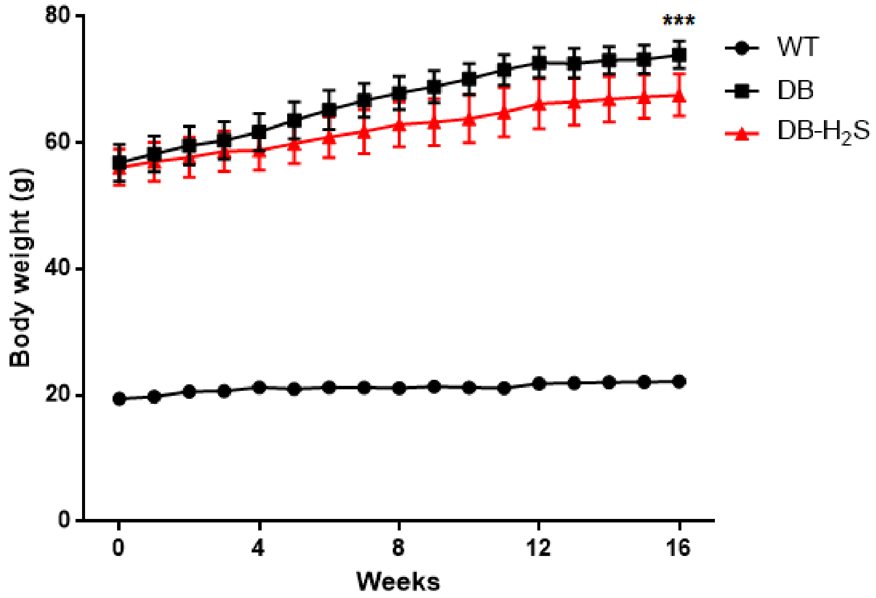

Figure S1 | Effects of exogenous H_2S on body weight. WT, wild type mouse group (n = 6); DB, db/db mouse group (n = 6); DB-H_2S, db/db mouse treated with NaHS group (n = 6). Data are shown as mean ± SEM. Differences were analyzed by one-way ANOVA with Tukey's multiple comparisons test and denoted as follows: *** P<0.001, WT vs. DB.

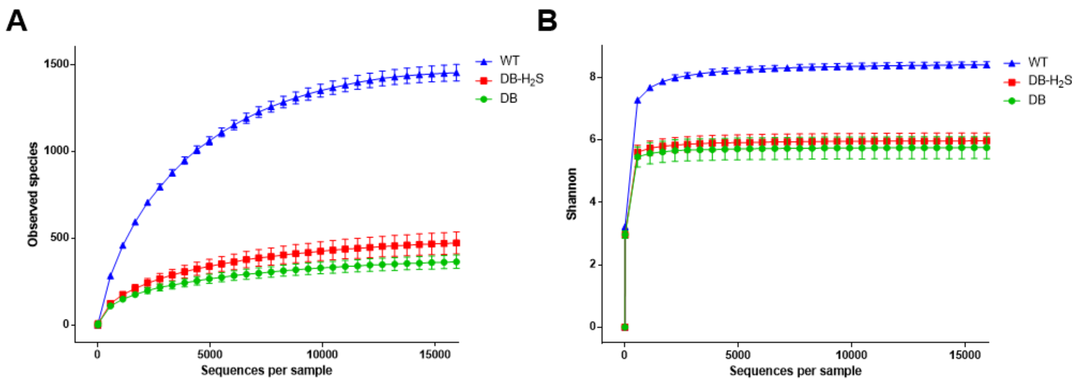

Figure S2 | Rarefaction curves of bacterial observed species and Shannon diversity for three groups. **(A)** Rarefaction curves of observed species from cecum samples of individual mice in WT group (blue), DB group (green), or DB-H_2S group (red); **(B)** Rarefaction curves of Shannon diversity in WT group (blue), DB group (green), or DB-H_2S group (red). WT, wild type mouse group (n = 6); DB, db/db mouse group (n = 6); DB-H_2S, db/db mouse treated with NaHS group (n = 6). Data are shown as mean ± SEM.

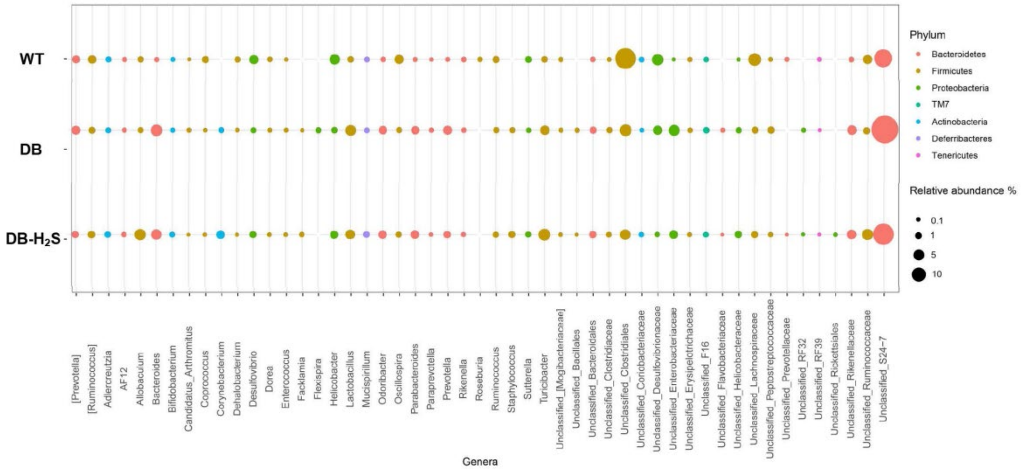

Figure S3 | Effect of exogenous H_2S on the gut microbiota composition. Prevalent bacterial genera were identified in at least one group relative abundance ≥ 0.1%, with relative abundance denoted by circle size and colors representing different phylum. WT, wild type mouse group (n = 6); DB, db/db mouse group (n = 6); DB-H_2S, db/db mouse treated with NaHS group (n = 6).

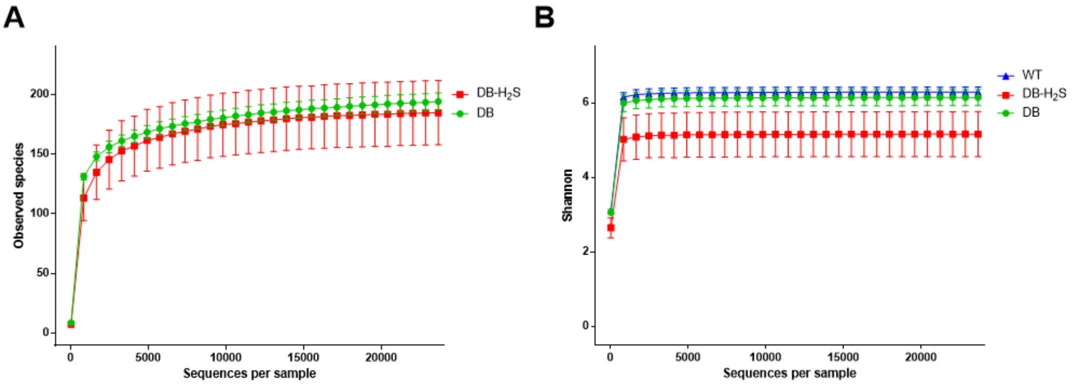

Figure S4 | Rarefaction curves of fungal observed species and Shannon diversity for three groups. **(A)** Rarefaction curves of observed species from cecum samples of individual mice in WT group (blue), DB group (green), or DB-H_2S group (red); **(B)** Rarefaction curves of Shannon diversity in WT group (blue), DB group (green), or DB-H_2S group (red). WT, wild type mouse group (n = 6); DB, db/db mouse group (n = 6); DB-H_2S, db/db mouse treated with NaHS group (n = 6). Data are shown as mean ± SEM.

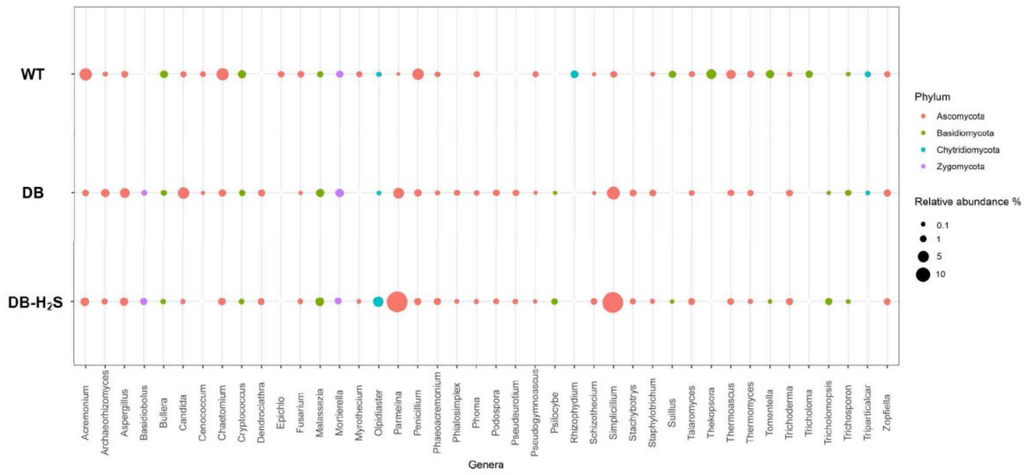

Figure S5 | Effect of exogenous H_2S on the gut mycobiome composition. Prevalent fungal genera were identified in at least one group relative abundance ≥ 0.5%, with relative abundance denoted by circle size and colors representing different phylum. WT, wild type mouse group (n = 6); DB, db/db mouse group (n = 6); DB-H_2S, db/db mouse treated with NaHS group (n = 6).
